# Rhein protects against renal aging and fibrotic injury by multiple targets through inhibition of TNF-α-mediated autophagy and necroptosis crosstalk

**DOI:** 10.3389/fphar.2026.1693000

**Published:** 2026-01-26

**Authors:** Yue Tu, Wenjie Liu, Tianqing Sang, Liuyunxin Pan, Wei Wu, Qijun Fang, Yinglu Liu, Buhui Liu, Yigang Wan

**Affiliations:** 1 Department of Traditional Chinese Medicine Health Preservation, Acupuncture, Moxibustion and Massage College, Health Preservation and Rehabilitation College, Nanjing University of Chinese Medicine, Nanjing, China; 2 Institute of Chinese Medicine, Nanjing University, Nanjing, China; 3 Jiangsu Provincial Technology Engineering Research Center of TCM Health Preservation, Nanjing University of Chinese Medicine, Nanjing, China; 4 Department of Traditional Chinese Medicine, Nanjing Drum Tower Hospital, Affiliated Hospital of Medical School, Nanjing University, Nanjing, China; 5 Department of Anatomy, School of Basic Medical Sciences, Xuzhou Medical University, Xuzhou, China

**Keywords:** rhein, renal aging, renal fibrosis, autophagy, necroptosis, TNF-α

## Abstract

**Introduction:**

Rhein, an anthraquinone derived from rhubarb, exhibits renoprotective effects in aging and kidney injury; however, the mechanistic interplay with TNF-α-mediated cell death pathways remains undefined.

**Methods:**

Using D-galactose (D-gal)-treated NRK-52E cells and aged rats, we assessed rhein’s effects with/without mTOR regulators (rapamycin/MHY1485) or etanercept (TNF-α inhibitor). Protein levels of klotho, phosphorylated (p)-mTOR, p-p62, caspase-8, beclin1, light chain 3 II, receptor-interacting protein kinase (RIPK)1, RIPK3, and p-mixed lineage kinase-like (MLKL) were measured. The concentration of reactive oxygen species (ROS) and the staining for senescence-associated-β-galactosidase (SA-β-gal) were also assessed. The network framework of “rhein–target–pathways” was identified. Additionally, serum untargeted metabolomics and KEGG pathway analysis were performed to identify altered metabolites and underlying metabolic pathways.

**Results:**

The results indicated that rhein increased the protein levels of klotho, p-mTOR, p-p62, and caspase-8, as well as decreased the concentration of ROS, the staining for SA-β-gal, and the protein levels of beclin1, light chain 3 II, RIPK1, RIPK3, and p-MLKL in NRK-52E cells exposed to D-gal. Compared to mTOR regulators (rapamycin or MHY1485) alone, the co-treatment of rhein and mTOR regulators decreased mTOR-mediated autophagy signaling in NRK-52E cells treated by D-gal. In addition, rhein decreased tumor necrosis factor (TNF)-α and TNF-α receptor1 protein levels. Interestingly, the effects of etanercept in TNF-α-mediated necroptosis and autophagy were similar to those of rhein. Consistent with the in vitro findings, rhein alleviated oxidative stress and exerted renal protective effects in aged model rats. Serum untargeted metabolomic analysis revealed that rhein treatment significantly altered 85 metabolites compared to the model group rats, with 35 metabolites upregulated and 50 downregulated. KEGG pathway analysis identified the TNF-α signaling pathway as a key metabolic pathway involved.

**Discussion:**

In conclusion, rhein protected the kidneys by activating p-mTOR and downregulating TNF-α, necroptosis and autophagy. Rhein mitigates renal aging and fibrotic injury by targeting TNF-α-mediated autophagy-necroptosis crosstalk, positioning it as a novel multi-target therapeutic agent for age-related kidney injury.

## Introduction

Kidney disease represents a leading cause of mortality worldwide and poses a critical public health burden. Aging profoundly accelerates renal functional decline, characterized by structural pathologies including glomerulosclerosis, interstitial fibrosis, and tubular atrophy (Denic et al., 2016). Given the inevitability of age-related renal deterioration—often exacerbated by inflammation—the development of protective interventions for aging kidneys is urgently needed.

Rhein, an anthraquinone compound derived from the rhubarb plant (scientifically referred to as Radix et Rhizoma Rhei or colloquially as Da Huang in China), exhibits diverse pharmacological bioactivities such as anti-inflammatory, anti-fibrogenesis, anti-tumor, and anti-angiogenic properties (Cheng et al., 2021; Henamayee et al., 2020). Klotho is a well-known anti-aging protein available in membrane-bound or soluble forms (Wang and Zhao, 2024). It plays a crucial role in maintaining renal equilibrium and serves not only as a responsive biomarker but also as a promising therapeutic target for various kidney disorders (Kale et al., 2021). Bi et al. found that rhein can effectively preserve klotho in lipopolysaccharide (LPS)-induced acute inflammation and kidney injury by facilitating the degradation of Toll-like receptor 4 (TLR4) (Bi et al., 2018). Rhubarb, as a herbal medicine, is commonly used in clinical practice for treating kidney diseases (Kang et al., 1993; Zhang et al., 2015). Our previous study proved that rhubarb possesses the ability to mitigate renal fibrosis and autophagy in rats with adenine-induced kidney damage, and rhein can inhibit starvation-induced autophagy by regulating the AMP-activated protein kinase (AMPK)/mammalian target of rapamycin (mTOR) and p38/Erk mitogen-activated protein kinase (MAPK) signaling pathways, thereby contributing to its renoprotective activities (Tu et al., 2017). However, the multi-target mechanisms of rhein against renal aging and fibrotic injury, particularly regarding crosstalk between cell death pathways, remain unexplored.

Critically, autophagy and necroptosis exhibit dynamic crosstalk in programmed cell death (PCD) during renal decline (Erekat, 2022). Studies have shown that autophagy mainly involves the mTOR-mediated production of autophagosomes, and mTOR-dependent signaling is maintained for different aspects of cellular senescence to take place (Cayo et al., 2021; Huang et al., 2021). Autophagic dysfunction is marked by upregulation of beclin1 and the conversion of light chain 3 (LC3) I to LC3 II, alongside downregulation of p62, in the pathogenesis of various kidney diseases (Tang et al., 2020; Yang et al., 2022). Necroptosis is a form of PCD that critically depends on caspase-8, receptor-interacting protein kinase (RIPK) 1 and RIPK3, and mixed lineage kinase-like (MLKL), characterized by cellular changes typically associated with necrosis (Galluzzi et al., 2017). Inhibition of caspase-8 induces necroptosis by prompting RIPK1 and RIPK3 activation and subsequent phosphorylation of the pseudokinase MLKL. Tumor necrosis factor-α (TNF-α) is a critical proinflammatory cytokine, and its signaling pathway is involved in various inflammation-related diseases (Bradley, 2008). Necroptosis can be triggered by death receptors such as TNF-α receptor 1 (TNFR1), namely, RIPK1 can activate RIPK3 upon resistance to TNFR1 ligation (Galluzzi et al., 2017). Among various accelerated aging models, the D-galactose (D-gal)-induced aging model, which operates through reactive oxygen species (ROS) accumulation, is widely preferred due to its convenience, minimal side effects, and high survival rate (Azman and Zakaria, 2019). In neuroblastoma cell lines, D-gal can induce significant toxicity and viability loss. The underlying mechanisms involve not only necrotic cell death features but also autophagy activation, as indicated by punctate GFP-LC3 distribution. Li et al. proved that the necroptosis and autophagy inhibitors, not the caspase inhibitor, alleviate the toxic effect of D-gal, confirming that D-gal can induce autophagy and necroptosis in neuroblastoma cells (Li et al., 2011). Our previous study demonstrated that D-gal can induce aging in the kidneys and renal tubular cells by promoting LC3-I to LC3-II conversion and enhancing beclin1 protein levels, indicating enhanced renal autophagic activity (Liu et al., 2018).

In this study, a series of *in vitro* and *in vivo* experiments was designed to verify whether rhein ameliorates D-gal-induced renal aging and fibrotic injury. We hypothesized that its mechanism involves targeted binding to TNF-α, an inflammatory cytokine that serves as a molecular link between autophagy and necroptosis (Wu et al., 2021). Our findings demonstrated that the inhibition of TNF-α activation by rhein promoted mTOR signaling and mitigated autophagy and necroptosis crosstalk. Therefore, rhein has strong potential as a multi-target therapeutic agent for renal aging and fibrotic injury.

## Materials and methods

### Chemicals and reagents

D-gal used in this study was sourced from Sigma–Aldrich Chemical Co. (St Louis, MO, United states). A concentrated solution of D-gal was formulated at a dosage of 180 mg/mL by mixing 15.75 g D-gal with 87.5 mL of distilled water. Vitamin E (VE) *in vivo* and *in vitro* was procured from 2 different suppliers: Yuanye Bioscience Co., Ltd. (Shanghai, China) and Maibo Bioscience Co., Ltd. (Nanjing, China). Rhein was obtained from Yuanye Bioscience Co., Ltd. (Shanghai, China). Rapamycin and MHY1485 were acquired from Sigma–Aldrich Chemical Co. (St Louis, MO, United states). Etanercept was supplied by AbMole inhibitor leader (Houston, TX, United states).

### Cell cultivation and experimental intervention

NRK-52E cells, a rat kidney epithelial cells, were provided by Dr. Jian Yao from the University of Yamanashi. We cultured NRK-52E cells, at 33 °C in 5% CO_2_ in Dulbecco’s modified Eagle’s medium/Ham’s F-12 (HyClone) containing 5% fetal bovine serum (FBS; Gibco, Grand Island, NY, United States). On the basis of a previous study (Liu et al., 2018), 100 mM D-gal treatment for 24 h was used to induce cellular aging in NRK-52E cells. The cells were exposed to D-gal and then co-treated with rhein (1, 3, and 5 μg/mL), VE (15, 25, and 50 μg/mL), rapamycin (100 μM), MHY1485 (2 μM), or etanercept (2 μg/mL) in different experiments to investigate the effect of rhein on cellular aging and determine the underlying mechanisms.

### Animal model and experimental design

We purchased male Sprague–Dawley (SD) rats, each with a mass of around 200 g, from SPF (Beijing) Biotechnology Co., Ltd. [License No: SYXK (SU) 2019-0058]. The protocol for the present research was approved by the Nanjing University Medical School’s Animal Ethics Committee with permit number 2020AE02026. This study is reported in accordance with ARRIVE guidelines. All methods were conducted in accordance with relevant guidelines and regulations. The animals were maintained in a pathogen-free environment with controlled temperature (22 °C with a variance of 3 °C) and humidity (50% with a tolerance of 10%), following a 12-h alternating light and dark schedule. They were provided with standard diet pellets and unrestricted access to water at the Experimental Animal Center of Nanjing Drum Tower Hospital, which is affiliated with Nanjing University Medical School.

Before the renal aging and fibrotic injury models were established, the SD rats were acclimated to the animal facilities for 1 week. The rats were randomly divided into seven groups: Normal group, D-gal (Model) group, D-gal + low dose of rhein (Rhein-low) group, D-gal + high dose of rhein (Rhein-high) group, D-gal + Vitamin E (VE) group, D-gal + rapamycin (Rapamycin) group, and D-gal + MHY1485 (MHY1485) group. The rats in the Normal group were given sham operation, whereas the rats in the other groups were given unilateral nephrectomy under isoflurane anesthesia. A week after the operations, the rats in the Normal group were given distilled water for a duration of 8 weeks, whereas the rats in the other groups were given D-gal at a daily dosage of 300 mg/kg via intraperitoneal injection and different drugs by oral administration. The rats in the Rhein-low group were given rhein solution at a daily dosage of 50 mg/kg, the rats in the Rhein-high group were given rhein solution at a daily dosage of 100 mg/kg, the rats in the VE group were given VE solution at a daily dosage of 30 mg/kg for 8 weeks, the rats in the Rapamycin group were given 1 mg/kg/day rapamycin solution for 4 weeks, and the rats in the MHY1485 group were given 1 mg/kg/day MHY1485 for 4 weeks. The weight of the rats was collected at 0, 2, 4, 6, and 8 weeks. At the end of the 8-week study, all rats were euthanized. They were anesthetized using isoflurane and sacrificed by cardiac puncture. Following this, blood serum and kidney tissues were collected to detect various indicators.

### Cell viability assessment

Cell viability was determined using CCK-8 assay (Biosharp, Shanghai, China), following the protocol provided by the manufacturer. The cells were plated into 96-well plates at a concentration of 0.5 × 10^4^ cells per well, with a volume of 100 mL of growth medium per well. After incubating the cells for the specified period, 10 mL of CCK-8 reagent was introduced into each well, and the plates were then incubated for an additional 2 h at 37 °C. The optical density of each well was measured at a wavelength of 450 nm using a spectrophotometer, and the cell viability was calculated. At least three parallel measurements were performed for each of the experiments.

### Western blot

Western blot (WB) analysis was carried out as previously described (Liu et al., 2021; Tu et al., 2014). The levels of the phosphorylated and total proteins of mTOR and the phosphorylated and total proteins of p62, beclin1, LC3, RIPK1, RIPK3, and caspase-8 were assessed using anti-phospho mTOR (Ser2448; 1:1000, #5536), anti-mTOR (1:1000, #2983), anti-phospho p62 (1:1000, #39786), anti-p62 (1:1000, #23214), anti-beclin1 (1:1000, #3495), anti-LC3A/B (1:1000, #12741), anti-RIPK1 (1:1000, #3493), anti-RIPK3 (1:1000, #15828), anti-caspase-8 (1:1000, #4790) and anti-cleaved-caspase-8 (1:1000, #8592) antibodies, respectively (Cell Signaling, Beverly, MA, United States). The levels of klotho and the phosphorylated protein of MLKL were assessed using anti-klotho (1:1000, PA5-88303) and anti-phospho MLKL (Ser358; 1:1000, PA5-105678) antibodies, respectively (Invitrogen, United States). The total protein level of MLKL was determined using anti-MLKL (1:2000, ab243142) antibody (Abcam, Cambridge, MA, United States). TNF-α, TNFR1, p53 and p21 levels were evaluated with anti-TNF-α (1:1000, abs149748), anti-TNFR1 (1:1000, abs130218), anti-p53 (1:1000, abs127784) and anti-p21 (1:1000, abs149756) antibodies, respectively (Absin Bioscience, Shanghai, China). Glyceraldehyde-3-phosphate dehydrogenase (GAPDH) protein level was detected by an anti-GAPDH (1:3000, #5174) antibody (Cell Signaling, Beverly, MA, United States) as a loading control. The immunoblots were developed using an enhanced chemiluminescence detection system (Tanon-5200Muilti, Shanghai, China), and the intensity of the bands was analyzed using densitometry with Image J Software (NIH, US, http://rsbweb.nih.gov/ij/index.html).

### Senescence-associated-β-galactosidase staining assay

The senescence detection was conducted using the senescence-associated (SA)-β-gal Staining Kit (Beyotime, Shanghai, China) following the provided protocol. After treating with the corresponding drugs, the medium was replaced with β-galactosidase staining solution for cell fixation for 15 min. Subsequently, the cells were immersed in the staining solution and incubated overnight. For tissue analysis, kidney cryosections of 4 μm thickness were adhered to slides, treated with a mixture of 0.2% glutaraldehyde and 2% formaldehyde for fixation, then processed through the staining solution overnight. Senescent cells were characterized by their blue-green coloration. Representative images of the SA-β-gal staining in cells and tissue sections were obtained using a light microscope (Olympus, Tokyo, Japan) at ×200 magnification.

### ROS fluorescent staining

To measure the levels of ROS in NRK-52E cells and renal tissues, we used the fluorescent probe dihydroethidium (D7008, Sigma, United States). After treating with the appropriate medications, the growth medium was subsequently removed and the sample was treated with dihydroethidium solution for 30 min in the dark condition. The cryostat kidney sections were incubated in dihydroethidium solution for the similar time. A blue fluorescent dye, 2-(4-Amidinophenyl)-6-indolecarbamidine dihydrochloride (DAPI), was applied to stain DNA for a period of 10 min. Then, intracellular and tissue sections’ typical images of ROS were examined under a fluorescent microscope (Nikon Eclipse Ti2, Tokyo, Japan). The resultant images were recorded using a camera (Nikon DS-Ri2, Japan) at ×200 magnification.

### Immunofluorescence assay

NRK-52E cells transfected with pmRFP-LC3 using Lipofectamine 2000 (Invitrogen, Carlsbad, CA, United States) were exposed to D-gal and co-treated with the corresponding drugs. The typical confocal images of NRK-52E cells were subsequently acquired at ×200 magnification by using an Olympus CKX41-F32FL fluorescence microscope (Olympus, Tokyo, Japan).

### Identification of genes associated with aging and rhein target proteins

We utilized the GeneCards database (https://www.genecards.org/), HAGR database (https://genomics.senescence.info/), OMIM database (http://www.omim.org/), and DisGeNet database (http://www.disgenet.org/home/), focusing the genes related to the search term “aging.” The search results only retained the studies on *Homo sapiens*. Following this, we retrieved the SMILES structure of rhein from PubChem (https://pubchem.ncbi.nlm.nih.gov/) and employed SwissTarget (http://www.swisstargetprediction.ch/) to forecast its potential protein targets. Subsequently, we integrated the predicted targets of rhein with the aging-related genes using Venny software (https://bioinfogp.cnb.csic.es/tools/venny/), generating a Venn diagram to pinpoint the intersecting targets.

### Pathway prediction of rhein for aging

Target information was uploaded to the DAVID online analysis platform (https://david.ncifcrf.gov/) for the purpose of exploring signaling pathways. The analysis was conducted with *H. sapiens* as both the criterion and background, ensuring that the results are specific to human biology. The Kyoto Encyclopedia of Genes and Genomes (KEGG) databases were used to analyze pathways related to the targets (Kanehisa et al., 2025; Kanehisa and Goto, 2000). The P-value was utilized to gauge the level of enrichment, with a higher P-value signifying a greater likelihood of enrichment, and conversely, a lower P-value indicating a lesser chance. As a result, the aging-related pathways associated with rhein were chosen based on a significance threshold of P < 0.01. The visualization of the enrichment outcomes was accomplished using Cytoscape3.9.0 software.

### Molecular docking verification

A molecular docking study was conducted to investigate the possible binding affinities of rhein with proteins associated with aging. The three-dimensional models of target proteins were sourced from the RCSB database (https://www.rcsb.org/), and Open Babel software was utilized to transform these models into the mol2 format. Rhein and its prospective therapeutic counterparts were prepared and optimized for energy in Autodock Tools 1.5.7. Subsequently, the molecular docking simulations were executed using Autodock Vina 1.2.0.

### Biochemical analyses

In the Department of Laboratory Medicine of Nanjing Drum Tower Hospital, an automatic biochemical analyzer was employed to assess key renal function indicators, including blood urea nitrogen (BUN) and serum creatinine (Scr), as well as liver function markers such as alanine transaminase (ALT), aspartate transaminase (AST), and alkaline phosphatase (ALP). Serum TNF-α (#AF3056-A) and 8-hydroxydeoxyguanosine (8-OHdG) (#AF3100-A) levels were measured using specific enzyme-linked immunosorbent assay (ELISA) kits, purchased from Hunan Aifang Biotechnology Co., Ltd. (Changsha, China). The assays were conducted according to the manufacturer’s protocols.

### Kidney histology

Kidney cortex tissue samples were embedded in paraffin and sliced into 4 μm sections. The sections were dewaxed with xylene, washed with ethanol, and stained with Masson’s trichrome. The typical fibrosis area in kidney tissues was captured by using a light microscope at ×400 magnification.

### Serum untargeted metabolomics analysis

Frozen serum samples (−80 °C) from Model, Rhein-high and VE groups were thawed on ice. A 50 µL aliquot was mixed with 300 µL ice-cold extraction solution (ACN:Methanol = 1:4, V/V) containing internal standards in a 2 mL tube. After vortexing (3 min) and centrifugation (12,000 rpm, 10 min, 4 °C), 200 µL supernatant was incubated (−20 °C, 30 min) and recentrifuged (12,000 rpm, 3 min, 4 °C). Finally, 180 µL supernatant was analyzed by LC-MS.

Metabolite separation used a Waters ACQUITY UPLC BEH C18 column (1.8 µm, 2.1 × 100 mm, 40 °C) with a mobile phase of (A) 0.1% formic acid in water and (B) 0.1% formic acid in acetonitrile. The gradient (flow rate 0.4 mL/min) was: 5% B initial; to 90% B over 11 min; hold 90% B for 1 min; to 5% B in 0.1 min; re-equilibrate at 5% B for 1.9 min. The injection volume was 2 µL.

Data acquisition used information-dependent acquisition (IDA) mode (Analyst TF 1.7.1 software). Key source parameters: GAS1/GAS2: 50 psi; CUR: 35 psi; TEM: 550 °C (ESI+)/450 °C (ESI-); DP: ±60 V; ISVF: ±5,000/±4000 V. TOF-MS scans (50–1000 Da, 200 ms accumulation) used dynamic background subtraction. Product ion scans (25–1000 Da, 40 ms accumulation) used collision energy ±30 V (mode-dependent), CES 15, unit resolution, charge state 1, intensity threshold >100 cps, isotope exclusion (4 Da), mass tolerance 50 mDa, and ≤12 candidate ions/cycle.

Unsupervised principal component analysis (PCA) (unit-variance scaled data) was performed using the prcomp function in R (www.r-project.org). Differential metabolites between groups were identified using partial least squares discriminant analysis (PLS-DA)-derived variable importance in projection (VIP) score >1, |Log_2_FC| ≥1, and Student’s t-test P-value <0.05. Metabolites with >20% missing values within groups were excluded, which were analyzed using Venn diagrams. Results were visualized via cluster heatmaps and volcano plots. Metabolites were annotated against the KEGG Compound database and mapped to KEGG Pathways. The significance of the pathway enrichment was then assessed using a hypergeometric test; specifically, the analysis relied on unadjusted P-values.

### Immunohistochemistry

Following the immunohistochemistry protocol detailed by Tu et al. (Liu et al., 2018), renal tissue slides were subjected to overnight incubation at 4 °C with a primary anti-LC3 antibody (diluted 1: 500; Servicebio, China). Subsequently, they were treated with a horseradish peroxidase-linked anti-rabbit secondary antibody (1:500 dilution; Servicebio, China) for 10 min. The resultant kidney tissue alterations and areas of positive immunostaining were visualized under a light microscope at ×400 magnification.

### Transmission electron microscopic analysis

The samples (1 mm^3^) of kidney tissues were excised, fixed in 2.5% glutaraldehyde in PBS. Afterward, they were rinsed with 1 mmol/L phosphoric acid solution, followed by a secondary fixation using 1% osmium tetroxide. The tissue blocks were then sectioned to a thickness of 0.07 μm and subjected to staining with uranyl acetate and lead citrate. The ultrastructure of autophagosomes was examined under a transmission electron microscopy (JEM-1230, Tokyo, Japan) at ×2000 magnification.

### Statistical analysis

The statistical software SPSS 26.0 (IBM, Armonk, NY, United States) was utilized for data analysis. Data obtained from at least three independent biological replicates are expressed as the mean ± standard deviation (SD). For multiple comparisons, one-way analysis of variance (ANOVA) was conducted, followed by Tukey’s post-hoc test. P < 0.05 or <0.01 indicated a statistically significant difference.

## Results

### D-gal-induced renal aging and fibrotic injury were attenuated by rhein *in vitro*

**FIGURE 1:**
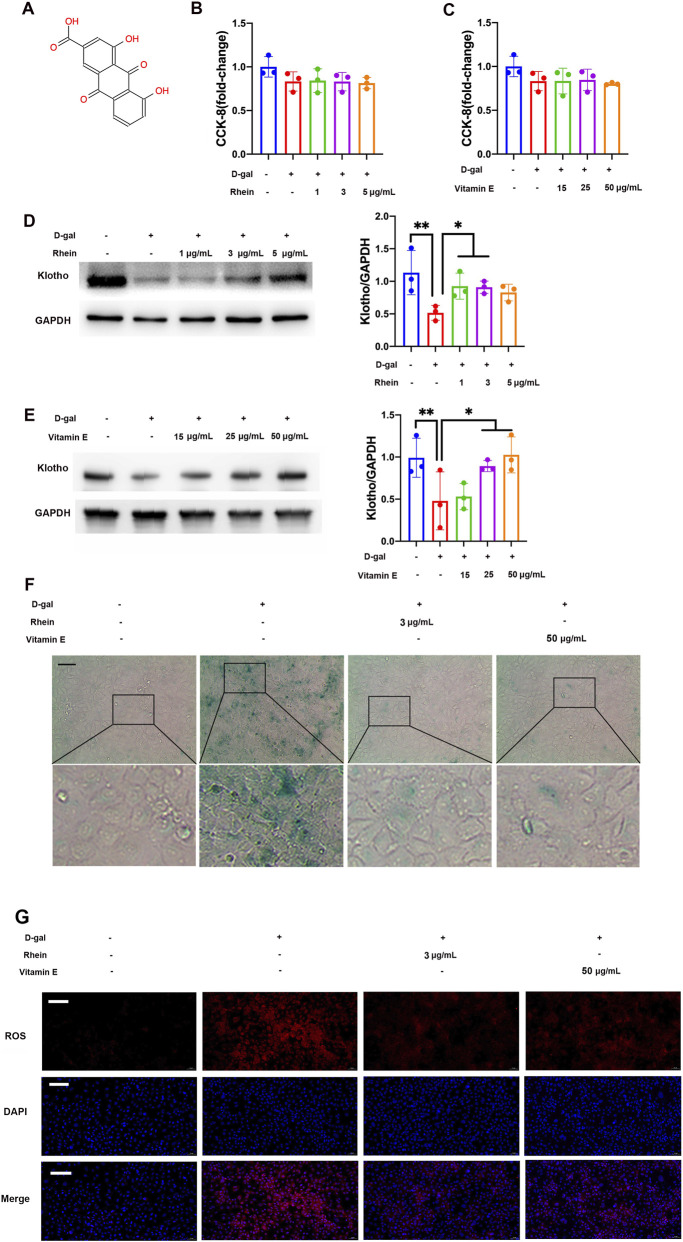
Effect of rhein on D-gal-treated NRK-52E cells’ viability and inhibition on D-gal-induced renal aging and fibrotic injury. **(A)** Molecular structural formula of rhein. **(B,C)** CCK-8 assay to determine the effect of rhein and VE on the viability of NRK-52E cells treated with D-gal. **(D,E)** WB analysis of klotho in the NRK-52E cells treated with D-gal alone or in combination with rhein or VE. **(F)** Typical images of SA-β-gal staining in the NRK-52E cells exposed to D-gal, with or without the addition of rhein or VE. **(G)** Typical images of ROS fluorescent staining in the NRK-52E cells exposed to D-gal and with the co-treatment of rhein or VE. Scale bar: 50 μm. Data are presented as mean ± SD (n = 3), *P < 0.05, **P < 0.01. Abbreviations: D-gal, D-galactose; ROS, Reactive oxygen species; SA-β-gal, Senescence-associated-β-galactosidase; VE, Vitamin E; WB, Western blot.

Figure 1A presents the molecular structure of rhein. Cellular viability was assessed via CCK-8 assay. The NRK-52E cells exposed to 100 mM D-gal and co-treated with rhein (1, 3, and 5 μg/mL) or VE (15, 25, and 50 μg/mL) for 24 h did not affect the cellular viability significantly (Figures 1B,C). Furthermore, the level of klotho protein expression was significantly downregulated in D-gal-treated NRK-52E cells for 24 h compared with that in the control group. However, co-treatment of rhein (1, 3, and 5 μg/mL) or VE (15, 25, and 50 μg/mL) improved the level of klotho protein expression in a dose-dependent manner (Figures 1D,E). Considering cellular viability and klotho protein expression, the concentrations of 3 μg/mL rhein and 50 μg/mL VE were used in the subsequent experiment. Similar results were obtained by immunostaining, which showed that 100 mM D-gal for 24 h increased SA-β-gal activity and ROS fluorescent staining in the NRK-52E cells. By contrast, SA-β-gal activity and ROS level were decreased for the NRK-52E cells subjected to D-gal and concurrently administered rhein (3 μg/mL) or VE (50 μg/mL) for 24 h (Figures 1F,G).

### Autophagy was reduced by rhein through mTOR signaling *in vitro*

**FIGURE 2:**
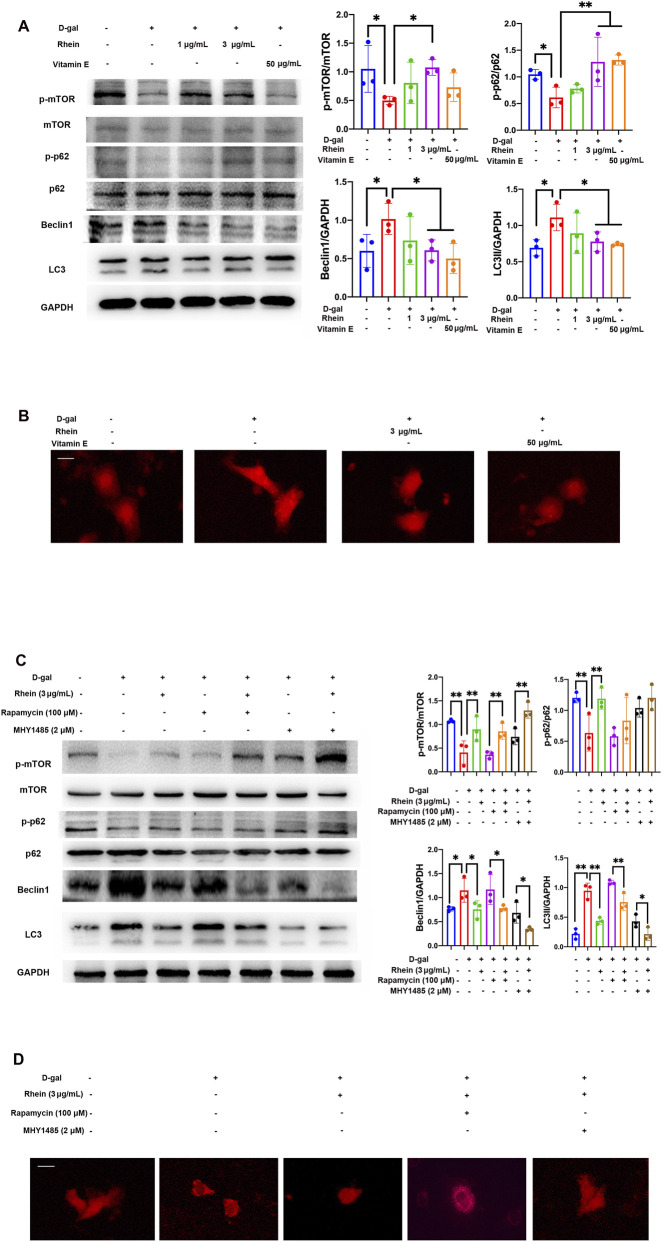
Rhein reduced autophagy through mTOR signaling in NRK-52E cells treated with D-gal. **(A)** WB analysis of p-mTOR, mTOR, p62, p-p62, beclin1, and LC3 in cultured NRK-52E cells exposed to 100 mM D-gal with or without rhein (1 and 3 μg/mL) and VE (50 μg/mL) treatment for 24 h **(B)** NRK-52E cells were transfected with pmRFP-LC3, treated with D-gal (100 mM) with or without rhein (3 μg/mL) and VE (50 μg/mL) treatment for 24 h, after which fluorescence microscopy was performed. Scale bar: 5 μm. **(C)** WB analysis of p-mTOR, mTOR, p-p62, p62, beclin1, and LC3 in cultured NRK-52E cells treated with 100 mM D-gal and rhein (3 μg/mL), rapamycin (100 μM), and MHY1485 (2 μM) for 24 h. **(D)** NRK-52E cells were transfected with pmRFP-LC3; treated with D-gal (100 mM) along with rhein (3 μg/mL), rapamycin (100 μM), and MHY1485 (2 μM) for 24 h, and then analyzed using fluorescence microscopy. Scale bar: 5 μm. Results are presented as the mean ± SD (n = 3). *P < 0.05, **P < 0.01. Abbreviations: D-gal, D-galactose; p-mTOR, phosphorylated mTOR; p-p62, phosphorylated p62; WB, Western blot.

The role of autophagic activity is vital in stress response and longevity (Wong et al., 2020). mTOR signaling is a central regulator of autophagy, and a growing list of evidence suggests that mTOR signaling influences aging and cellular senescence (Weichhart, 2018). In Figure 2A, WB analysis showed that rhein treatment increased phosphorylated-mTOR (p-mTOR) and phosphorylated-p62 (p-p62) protein levels that were downregulated due to D-gal stimulation in NRK-52E cells. Furthermore, the protein levels of beclin1 and LC3 II decreased with the treatment of rhein but were upregulated due to D-gal stimulation in NRK-52E cells. VE treatment had the mimic effect of increasing mTOR signaling and inhibiting autophagy. Consistent with the above results, D-gal led to the formation of numerous punctate dots in the NRK-52E cells that had been transfected with the pmRFP-LC3 construct. However, the number of these D-gal-induced LC3-positive puncta was significantly reduced upon cotreatment with either rhein or VE (Figure 2B). To elucidate the involvement of mTOR signaling in the inhibitory effect of rhein on autophagic activity, rapamycin (mTOR inhibitor) and MHY1485 (mTOR agonist) were co-treated with D-gal in the presence or absence of rhein in NRK-52E cells. Compared with rapamycin and MHY1485 alone, the protein level of p-mTOR was increased and the levels of autophagy signaling was decreased more obviously in the co-treatment of rhein in NRK-52E cells exposed to D-gal (Figure 2C). In Figure 2D, similar changes in LC3-labeled punctate dots were observed in NRK-52E cells treated with D-gal and co-treated with either rhein combined with rapamycin or MHY1485.

### Necroptosis was inhibited by rhein *in vitro*

**FIGURE 3:**
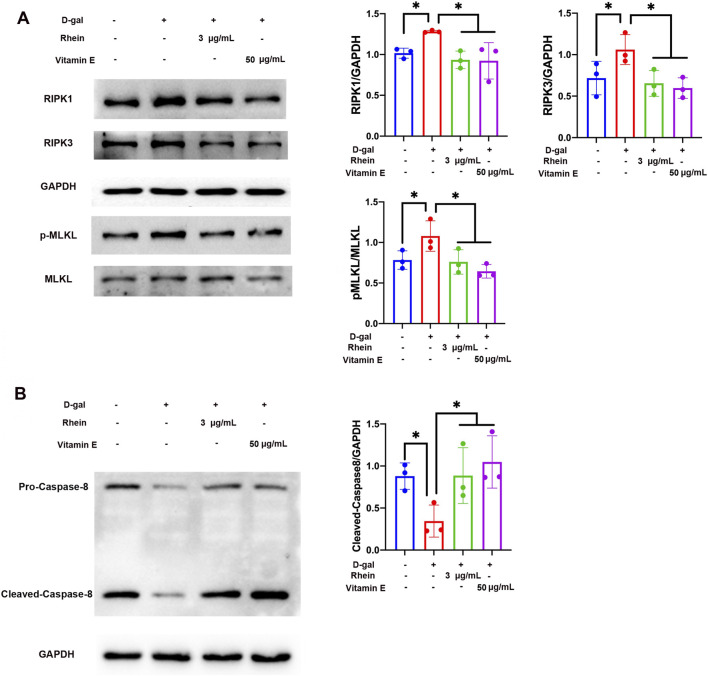
Rhein inhibited necroptosis in D-gal-treated NRK-52E cells. **(A)** WB analysis of RIPK1, RIPK3, p-MLKL, and MLKL in cultured NRK-52E cells exposed to 100 mM D-gal in the presence or absence of rhein (3 μg/mL) and VE (50 μg/mL) treatment for 24 h **(B)** WB analysis of pro- and cleaved-caspase-8 in cultured NRK-52E cells exposed to 100 mM D-gal in the presence or absence of rhein (3 μg/mL) and VE (50 μg/mL) treatment for 24 h. Results are presented as the mean ± SD (n = 3). *P < 0.05. Abbreviations: D-gal, D-galactose; p-MLKP, phosphorylated MLKL; WB, Western blot.

Besides autophagy, necroptosis is another important mechanism of the cell death signaling pathway. D-gal-damaged cells show the characteristics of necrotic cell death; furthermore, the toxicity of D-gal can be mitigated by the necroptosis inhibitor necrostatin and autophagy inhibitor 3-methyladenine (Li et al., 2011). Phosphorylation of MLKL (p-MLKL) and MLKL oligomers, as well as RIPK1 and RIPK3, is a marker of necroptosis. As shown in Figure 3A, WB analysis revealed that rhein and VE treatment decreased the RIPK1, RIPK3, and p-MLKL protein levels that were upregulated due to D-gal stimulation in NRK-52E cells. Caspase-8, as a molecular switch, is an initiator caspase of death receptor-induced apoptosis and inhibits RIPK3-MLKL-dependent necroptosis (Fritsch et al., 2019). Figure 3B shows that rhein and VE treatment increased the protein level of caspase-8 that was downregulated due to D-gal stimulation in NRK-52E cells.

### Network pharmacology and molecular docking verification were employed to identify rhein’s potential anti-aging targets

**FIGURE 4:**
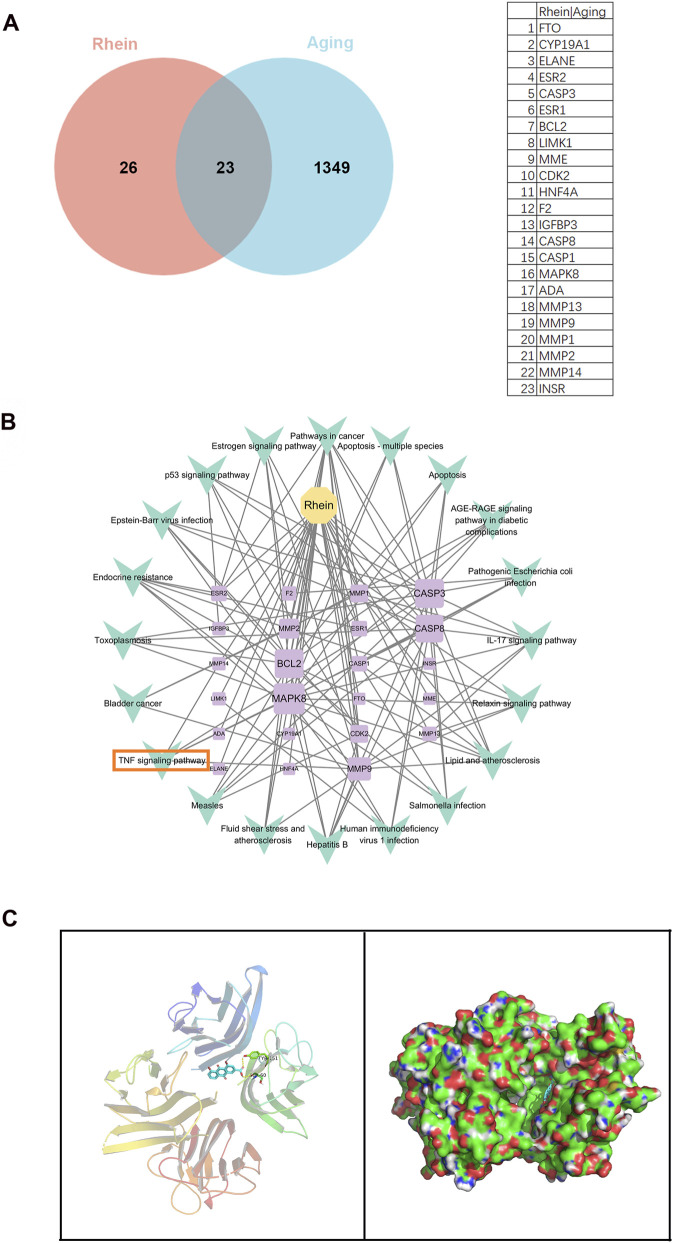
Investigative findings on the role of rhein in aging via network pharmacology. **(A)** A Venn diagram representing the overlap of 23 potential targets associated with both “rhein” and “aging.” **(B)** A graphical representation of the interactive network linking “rhein–target–pathway.” **(C)** Schematic illustrations of the molecular docking simulations showing the interaction between rhein and TNF-α.

Through data mining across GeneCards, HAGR, OMIM, and DisGeNet, a comprehensive list of 1,349 aging-related targets were retrieved. In parallel, the PubChem and SwissTarget databases were queried to identify 26 rhein targets, which were then cross-referenced with the aging targets to pinpoint 23 shared genes that may mediate rhein’s anti-aging effects. These intersections are depicted in Figure 4A, where the Venn diagram and an accompanying table highlight the convergence of rhein and aging targets. Further analysis using DAVID-KEGG was performed to uncover the key pathways associated with these targets, revealing that the TNF-α signaling pathway plays a crucial role in the “rhein–target–pathway” network, as illustrated in Figure 4B. The molecular docking studies, visualized in Figure 4C, demonstrate rhein’s interaction with TNF-α, with a calculated docking score of −8.4 kcal/mol, indicating a strong binding affinity, as detailed in Supplementary Table S1.

### TNF-α-mediated necroptosis and autophagy crosstalk were regulated by rhein in aged tubular cells *in vitro*

**FIGURE 5:**
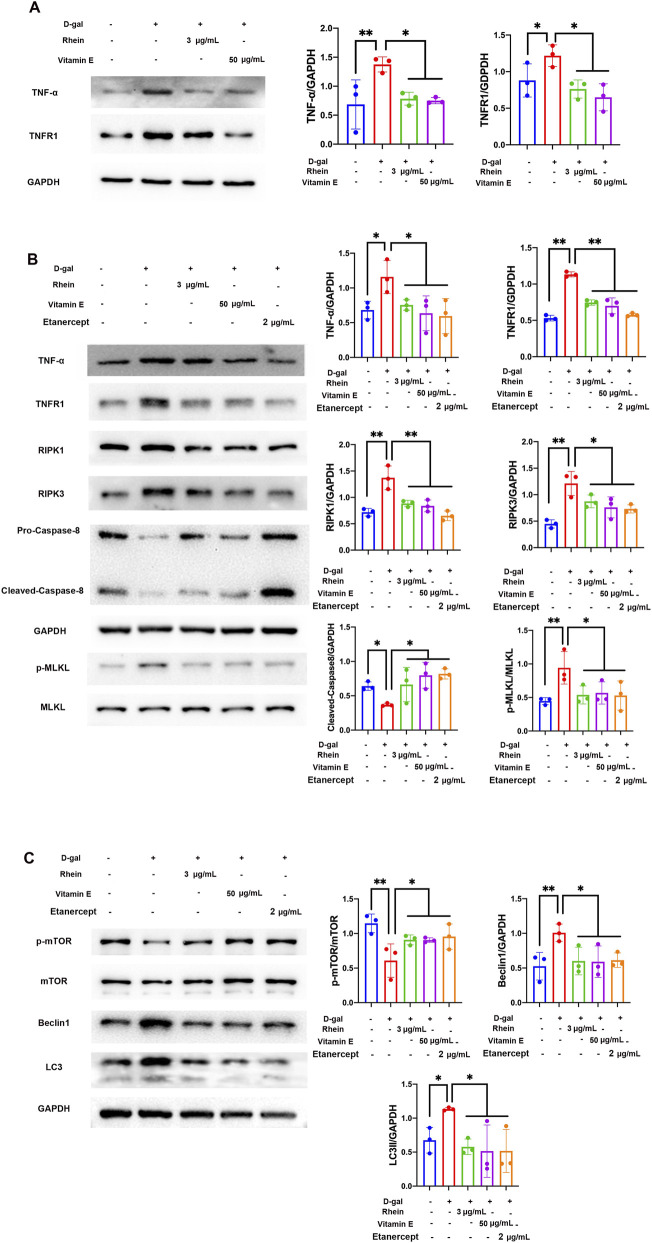
Rhein regulated the TNF-α-mediated necroptosis crosstalk in D-gal-treated NRK-52E cells. **(A)** WB analysis of TNF-α and TNFR1 in cultured NRK-52E cells exposed to 100 mM D-gal with or without rhein (3 μg/mL) and VE (50 μg/mL) treatment for 24 h. **(B)** WB analysis of TNF-α, TNFR1, RIPK1, RIPK3, pro-caspase-8, cleaved-caspase-8, p-MLKL, and MLKL in cultured NRK-52E cells exposed to 100 mM D-gal in the presence or absence of rhein (3 μg/mL), VE (50 μg/mL), and etanercept (2 μg/mL) treatment for 24 h. **(C)** WB analysis of p-mTOR, mTOR, beclin1, and LC3 in cultured NRK-52E cells exposed to 100 mM D-gal in the presence or absence of rhein (3 μg/mL), VE (50 μg/mL), and etanercept (2 μg/mL) treatment for 24 h. Results are presented as the mean ± SD (n = 3). *P < 0.05, **P < 0.01. Abbreviations: D-gal, D-galactose; TNFR1, tumor necrosis factor receptor-1; p-mTOR, phosphorylated mTOR; WB, Western blot.

Through network pharmacology analysis, we verified the TNF-α signaling pathway in D-gal-induced aged tubular cells by rhein. WB analysis showed that rhein and VE treatment decreased the TNF-α and TNFR1 protein levels that were increased due to D-gal stimulation in NRK-52E cells (Figure 5A). Moreover, etanercept, a TNF antagonist, similar to rhein and VE, could downregulate the TNF-α, TNFR1, RIPK1, RIPK3, and p-MLKL protein levels that were upregulated due to D-gal stimulation in NRK-52E cells (Figure 5B). Compared with the control, after treatment with rhein, VE, and etanercept, the protein level of caspase-8, an inhibitor of RIPK3-MLKL-dependent necroptosis, was upregulated in the NRK-52E cells stimulated by D-gal (Figure 5B). In addition, rhein, VE, and etanercept increased p-mTOR protein levels that were decreased due to D-gal stimulation but restored beclin1 and LC3II protein levels that were increased due to D-gal treatment (Figure 5C). In sum, rhein was found to regulate the crosstalk between TNF-α-mediated necroptosis and autophagy in aged tubular cells. These results suggest that rhein can modulate the interplay between necroptosis and autophagy, potentially providing protective effects in aged cells.

### D-gal-induced renal aging and fibrotic injury were attenuated by rhein *in vivo*

**FIGURE 6:**
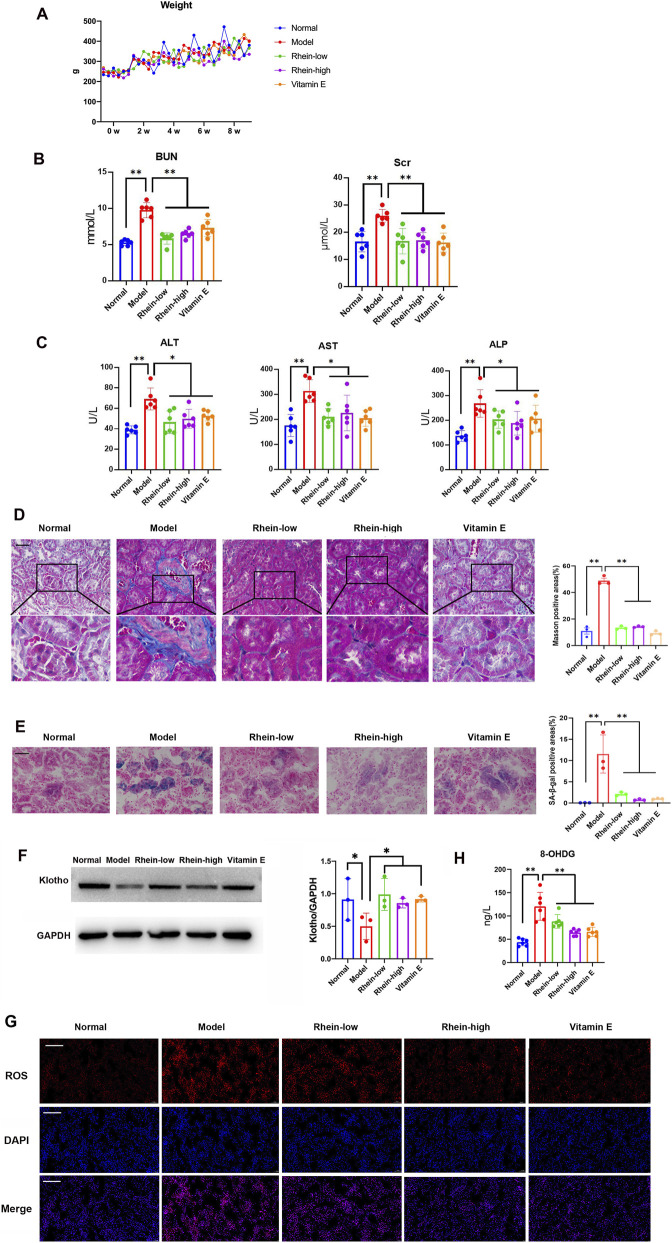
Rhein attenuated D-gal-induced renal aging and fibrotic injury in rats. **(A)** Body weight of the rats at 0, 2, 4, 6, and 8 weeks in the Normal, Model, Rhein-low, Rhein-high, and VE groups. **(B,C)** Renal and liver functions of BUN, Scr, ALT, AST, and ALP levels in the Normal, Model, Rhein-low, Rhein-high, and VE groups. **(D,E)** Masson and SA-β-gal staining of kidneys of rats, and statistical results in the Normal, Model, Rhein-low, Rhein-high, and VE groups. Scale bar: 20 μm. **(F)** WB analysis of klotho in kidneys in the Normal, Model, Rhein-low, Rhein-high, and VE groups. **(G)** Typical images of ROS fluorescent staining of kidneys of rats in the Normal, Model, Rhein-low, Rhein-high, and VE groups. Scale bar: 50 μm. **(H)** Measurement of 8-OHdG in the serum of rats in the Normal, Model, Rhein-low, Rhein-high, and VE groups. Results are presented as the mean ± SD (**(B,C,H)** n = 6; **(D,E,F)** n = 3). *P < 0.05, **P < 0.01. Abbreviations: ALT, Alanine Transaminase; AST, Aspartate Transaminase; ALP, Alkaline phosphatase; BUN, Blood urea nitrogen; D-gal, D-galactose; ROS, Reactive oxygen species; Scr, Serum creatinine; SA-β-gal, Senescence-associated β-galactosidase; VE, Vitamin E; W, Week; WB, Western blot; 8-OHdG, 8-hydroxydeoxyguanosine.

The effect of rhein on counteracting renal aging and fibrotic injury was subsequently confirmed in D-gal-induced model rats. In Figure 6A, the body weights of the rats in the Normal, Model, Rhein-low, Rhein-high, and VE groups rose gradually during the experimental process. By the 8th week, there was no notable variation in weight among these groups. The BUN, Scr, ALT, AST, and ALP levels of the rats in the Model group increased significantly in comparison with those in the Normal group. Treatment with rhein, at both low and high doses, along with VE, led to a significant reduction in these biochemical parameters in the treated rats compared to the Model group (Figures 6B,C). As shown in Figures 6D,E, the histopathological changes and senescence marker in the renal cortices from all groups were analyzed upon histological examination using light microscopy after Masson’s trichrome and SA-β-gal staining. Abnormality was found in the Model group, which was characterized by accumulation of the extracellular matrix, interstitial fibrosis, and increased SA-β-gal positive area. However, histopathological damage and senescence markers to renal interstitium in the renal aging and fibrotic injury model rats were alleviated by treatment with Rhein-low, Rhein-high, and VE. Furthermore, evidence from WB showed a significant reduction of the klotho protein expression in the kidneys of the Model group. By contrast, the protein expression of klotho in the kidneys of the Rhein-low, Rhein-high, and VE groups increased (Figure 6F). By the observation of fluorescent staining, the ROS level in renal tissues was increased in the Model group, while the ROS accumulation was decreased by treatment with Rhein-low, Rhein-high, and VE (Figure 6G). Compared with the control group, the serum 8-OHdG level in the Model group was increased. However, the 8-OHdG level was decreased by treatment with Rhein-low, Rhein-high, and VE (Figure 6H).

### Serum untargeted metabolomics identified TNF-α signaling as a key pathway in rhein’s renal protection

**FIGURE 7:**
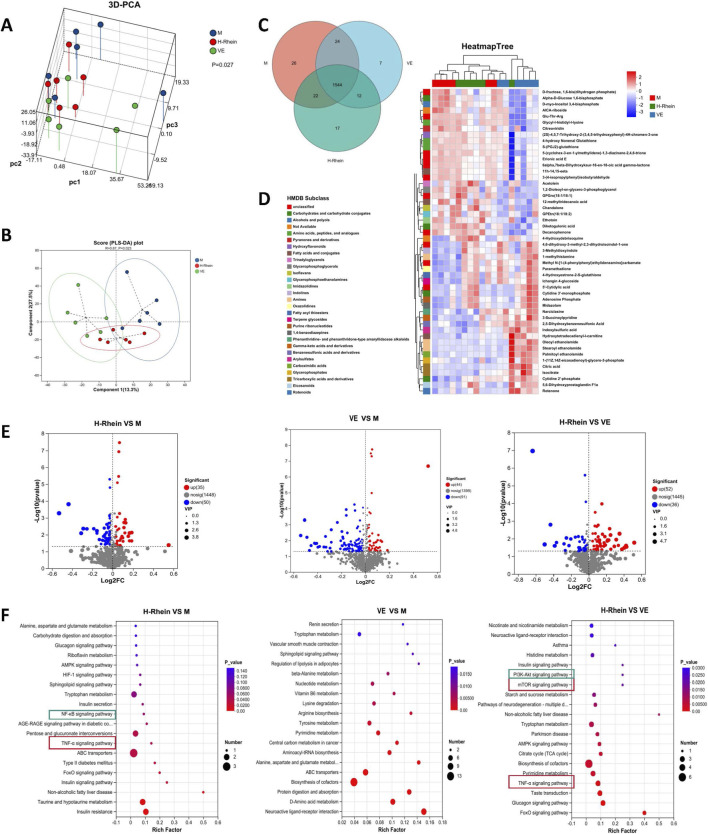
Regulation of serum metabolic profiles by rhein and pathway enrichment analysis. **(A)** PCA of metabolites in the Normal, Model (M), Rhein-high (H-Rhein), and VE groups. **(B)** PLS-DA of intergroup metabolites. **(C)** Venn diagram analysis of differential metabolites. **(D)** Heatmap of differentially abundant metabolites. **(E)** Volcano plot of differential metabolites. **(F)** KEGG pathway enrichment analysis of differential metabolites. The Rich Factor reflects the degree of enrichment, the dot size represents the number of differential metabolites, and the color gradient indicates the magnitude of the P-value (unadjusted), allowing for intuitive identification of significantly enriched biological pathways. Abbreviations: PCA, principal component analysis; PLS-DA, partial least squares-discriminant analysis; VE, Vitamin E.

Untargeted metabolomic analysis of peripheral blood serum from the Model, Rhein-high, and VE groups revealed distinct metabolic profiles. PCA and PLS-DA demonstrated clear separation among the three groups (Figures 7A,B). Venn analysis further indicated that both Rhein-high and VE interventions significantly altered serum metabolite levels (Figure 7C). Based on HMDB database enrichment analysis, Rhein-high primarily downregulated metabolites within categories such as carbohydrates and carbohydrate conjugates and amino acids, peptides, and analogues, while upregulating metabolites like amines and tricarboxylic acids and derivatives (Figure 7D). Quantitative analysis showed that compared to the Model group, Rhein-high treatment upregulated 35 metabolites and downregulated 50 metabolites. Compared to the VE group, Rhein-high upregulated 52 metabolites and downregulated 36 metabolites (Figure 7E). The top 20 significantly up- and down-regulated metabolites in each comparison are listed in Supplementary Table S2. KEGG pathway enrichment analysis indicated that differential metabolites between the VE and Model groups were mainly enriched in pathways like ABC transporters and cofactor biosynthesis. Notably, among the top 20 enriched pathways for differential metabolites in the Rhein-high group compared to both the Model and VE groups, the key signaling pathways including TNF-α signaling and its upstream/downstream regulators (NF-κB, mTOR, PI3K/AKT, AMPK) were commonly enriched. This suggests that Rhein-high may exert its biological effects by modulating these signaling pathways (Figure 7F).

### Renal aging and fibrotic injury were alleviated by rhein by TNF-α-mediated necroptosis and autophagy crosstalk *in vivo*

**FIGURE 8:**
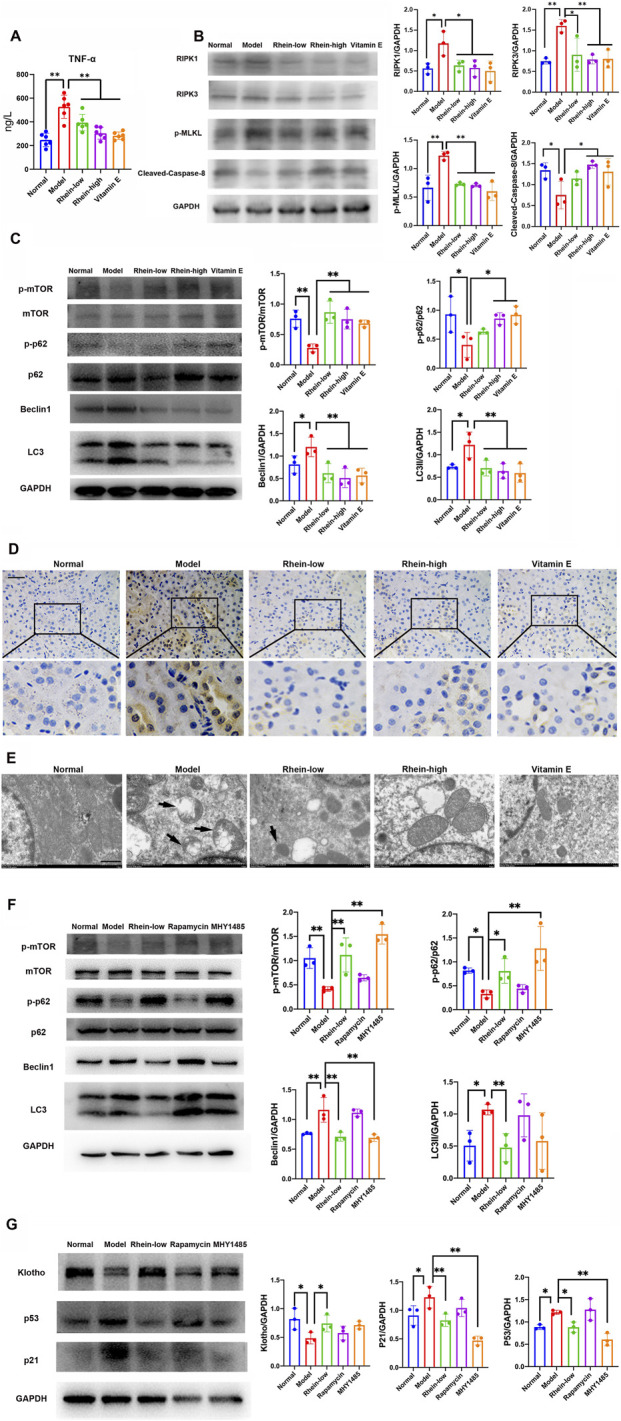
Rhein alleviated D-gal-induced renal aging and fibrotic injury by TNF-α-mediated necroptosis and autophagy in rats. **(A)** Measurement of serum TNF-α level of rats in the Normal, Model, Rhein-low, Rhein-high, and VE groups. **(B)** WB analysis of RIPK1, RIPK3, cleaved-caspase-8, and p-MLKL in kidneys in the Normal, Model, Rhein-low, Rhein-high, and VE groups. **(C)** WB analysis of p-mTOR, mTOR, p-p62, p62, beclin1, and LC3 in kidneys in the Normal, Model, Rhein-low, Rhein-high, and VE groups. **(D)** Typical images of immunohistochemical staining of LC3 in kidneys in the Normal, Model, Rhein-low, Rhein-high, and VE groups. Scale bar: 20 μm. **(E)** The quantity changes of autophagosome in kidneys in the Normal, Model, Rhein-low, Rhein-high, and VE groups by transmission electron microscopy. Scale bar: 1 μm. The black arrows indicate autophagosomes, which are distinctive for their double-membrane structure. **(F)** WB analysis of p-mTOR, mTOR, p-p62, p62, beclin1, and LC3 in kidneys in the Normal, Model, Rhein-low, Rapamycin, and MHY1485 groups. **(G)** WB analysis of klotho, p53, and p21 in kidneys in the Normal, Model, Rhein-low, Rapamycin, and MHY1485 groups. Results are presented as the mean ± SD (n = 3). *P < 0.05, **P < 0.01. Abbreviations: D-gal, D-galactose; TNFR1, tumor necrosis factor receptor-1; p-mTOR, phosphorylated mTOR; p-p62, phosphorylated p62; WB, Western blot.

Consistent with *in vitro* findings where rhein could prevent D-gal-induced renal aging and fibrotic injury, the key molecules in TNF-α-mediated necroptosis and autophagy in kidneys of D-gal-induced model rats were reversed by rhein treatment. ELISA result showed higher serum TNF-α level in D-gal-induced model rats, while this circulating level decreased by treatment with Rhein-low, Rhein-high, and VE (Figure 8A). Moreover, Rhein-low, Rhein-high, and VE could downregulate the RIPK1, RIPK3, and p-MLKL protein levels that were upregulated in D-gal-induced model rats; while the above treatment could increase the cleaved-caspase-8 protein level that was downregulated in the model rats (Figure 8B). WB analysis showed that Rhein-low, Rhein-high, and VE increased the levels of p-mTOR and p-p62 protein expression in D-gal-induced model rats (Figure 8C). By contrast, Rhein-low, Rhein-high, and VE decreased the protein expression levels of beclin1 and LC3 II in D-gal-induced model rats. Furthermore, immunohistochemical staining of LC3 demonstrated that autophagic specific marker increased in D-gal-induced model rats and decreased after the treatment with Rhein-low, Rhein-high, and VE (Figure 8D). Transmission electron microscopy revealed an increased number of typical autophagosomes in the D-gal-induced model rats, whereas treatment with Rhein-low, Rhein-high, and VE decreased the number of autophagosomes (Figure 8E). In addition, mTOR inhibitor and agonist, namely, rapamycin and MHY1485, were used to confirm the role of mTOR signaling in D-gal-induced model rats. As shown in Figures 8F,G, the results of WB revealed that MHY1485 obviously increased p-mTOR, p-p62, and klotho protein levels and decreased beclin1, LC3 II, p53, and p21 levels compared with those in the Model group. However, rapamycin did not change mTOR-mediated autophagy signaling in D-gal-induced model rats.

## Discussion

**FIGURE 9:**
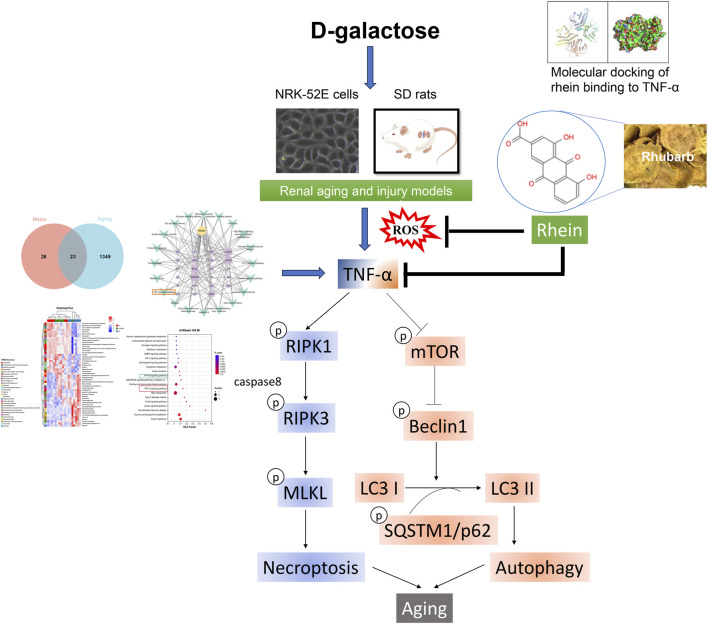
The schematic diagram of the mechanism in rhein targeting TNF-α-mediated autophagy and necroptosis in D-gal-induced renal aging and fibrotic injury by multiple targets.

The primary outcome of the present research is that the renoprotective effect of rhein in oxidative stress-induced renal aging and fibrotic injury was mediated by mTOR-mediated autophagy and RIPK1/RIPK3/MLKL-dependent necroptosis. Further exploration of aging and rhein based on network pharmacology analysis and the interaction between rhein and TNF-α might help identify the potential target for rhein. In addition, we demonstrated that rhein may target TNF-α-mediated autophagy and necroptosis in D-gal-induced renal aging and fibrotic injury (Figure 9). Therefore, rhein might serve as a promising therapeutic agent for treating renal aging and fibrotic injury by multiple targets.

Besides naturally aging models, accelerated aging models are often used in scientific research because of their benefits of short duration and high survival rate. The D-gal-induced accelerated aging model is a well-known model. D-gal is an aldohexose, a reducing sugar that naturally exists in the body; however, under the catalysis of galactose oxidase, it might be converted into aldose and hydroperoxide, resulting in the formation of ROS and subsequently causing multiple pathophysiological mechanisms, such as oxidative stress, inflammation, autophagy, and apoptosis (Azman and Zakaria, 2019; Alejandro, 2022). Renal function is usually assessed by measuring BUN and Scr, whereas liver function is usually assessed by ALT, AST, and ALP. According to Mo et al., in the D-gal administration-induced aging model, D-gal can increase the levels of the above renal and liver function indexes abnormally and induce the visualization of glomerular and tubulointerstitial lesions (Mo et al., 2017). 8-OHdG serves as a sensitive biomarker of the occurrence of oxidative DNA damage. It provides a direct indication of the degree of oxidative DNA damage and the level of oxidative stress in the body (Bruner et al., 2000). In the current study, our findings indicated that D-gal administration decreased the protein expression of the anti-aging molecule klotho, and the SA-β-gal staining area and ROS level increased both *in vitro* and *in vivo*. Furthermore, D-gal administration significantly increased BUN, ALT, AST, ALP and 8-OHdG levels *in vivo*.

Rhein has been used by the Chinese people as a mild laxative agent and an astringent since ancient times. It exhibits diverse pharmacological activities, including nephroprotective, anti-fibrogenesis, anti-microbial, anti-inflammatory, and anti-angiogenic properties (Cheng et al., 2021; He et al., 2011; Wang et al., 2010). In the current study, we demonstrated that rhein and VE improved klotho protein expression, SA-β-gal staining, and ROS accumulation both *in vitro* and *in vivo* in a dose-dependent manner or in adequate concentrations. Masson’s trichrome staining revealed that rhein and VE alleviated the accumulation of the extracellular matrix and renal fibrosis and reduced the BUN, Scr, ALT, AST, ALP and 8-OHdG levels in D-gal-induced model rats.

mTOR is an evolutionary conserved serine–threonine kinase that senses and combines a variety of environmental and intracellular signals (Saxton and Sabatini, 2017). Autophagy, a self-degradative process, is typically induced under physiological stress to eliminate damaged organelles and restore cellular homeostasis (Cao et al., 2021). High mTOR expression promotes protein synthesis, but its overexpression inhibits autophagy (Weichhart, 2018; Liu and Sabatini, 2020). The autophagy and mTOR-mediated autophagy signaling pathways are the key causes of aging (Liu and Sabatini, 2020). Upregulating autophagy is a mechanism of delaying senescence (Gomez-Cambronero and Kantonen, 2014). However, some believe that autophagy is a double-edged sword because excess activation or suppression of autophagy can disrupt the homeostatic degradation of protein and organelles in eukaryotic cells and play a key role in stress-induced cell mortality (Martinet et al., 2009). In our previous study, rhein inhibited Hank’s balanced salt solution (HBSS)-induced LC3 conversion in a dose-dependent manner. In addition, rhein can inhibit autophagy by upregulating the mTOR signaling pathway (Tu et al., 2017). In the present study, rhein increased the mTOR signaling pathway and inhibited autophagy in D-gal-induced renal aging and fibrotic injury model *in vitro* and *in vivo*. To elucidate the function of mTOR in the inhibitory impact of rhein on autophagic activity, we administered rapamycin and MHY1485 in the co-treatment with rhein. In the co-treatment of rhein and rapamycin or MHY1485, mTOR signaling increased and autophagy was inhibited significantly; this result was in contrast to that obtained with rapamycin or MHY1485 alone *in vitro*. Similar mTOR-related autophagy signaling changes were observed in the MHY1485 group *in vivo*, but rapamycin alone did not induce significant changes, highlighting the complexity of biological processes in D-gal-induced renal aging and fibrotic injury.

Besides autophagy, necroptosis is another vital PCD modality characterized by necrosis and apoptosis (Wang et al., 2016). The core participants in the necroptosis pathway are RIPK1, RIPK3, and MLKL. Sequential activation of RIPK1 and RIPK3 leads to MLKL phosphorylation and oligomerization, resulting in cell membrane disruption and release of cellular components (Royce et al., 2019). Duan et al. reported that the inhibition of RIPK1 by necrostatin-1 reduces neuroinflammation and ameliorates postoperative cognitive dysfunction in D-gal-induced aged mice (Duan et al., 2018). Moreover, Li et al. found that D-gal can induce necroptosis and activate autophagy in neuroblastoma cells (Li et al., 2011). In our current study, D-gal could also induce RIPK1, RIPK3, and p-MLKL protein levels in NRK-52E cells and D-gal-induced model rats. With the treatment of rhein or VE, the above protein levels of necroptosis decreased. Loss of caspase-8 activity allows the activation of necroptosis, namely, caspase-8-dependent cleavage prevents necroptosis (Weinlich et al., 2017). Our results demonstrated that caspase-8 protein level was downregulated in D-gal-stimulated NRK-52E cells; further, after the treatment of rhein or VE, caspase-8 protein level notably increased. Thus, rhein may reduce necroptosis by caspase-8-dependent cleavage.

mTOR exists as two multimeric functional complexes, mTOR complex 1 (mTORC1) and mTOR complex 2 (mTORC2). mTORC1 is sensitive to rapamycin, whereas mTORC2 is not directly inhibited by it. The rapamycin-sensitive mTORC1 has been identified as an autophagy inhibitor, and increasing evidence indicates that it plays a precise role in regulating necroptosis (Xie et al., 2023). For instance, Cai et al. found that mTORC1 hyperactivation contributes to catecholamine-induced cardiomyocyte necroptosis by suppressing macroautophagy, and rapamycin pretreatment can mitigate this process by restoring autophagy and inhibiting the necroptotic pathway (Cai et al., 2024). However, biological systems are inherently complex. Recently, Lin et al. found that tuberous sclerosis complex 2 plays a crucial role in modulating tumor susceptibility by influencing mTORC2 activity, with its absence leading to enhanced necroptosis (Lin et al., 2023). Thus, both mTORC1 and mTORC2 may have significant relationships with necroptosis. The anti-phospho mTOR (Ser2448; Cell Signaling, #5536) antibody used in this study to detect p-mTOR protein expression cannot differentiate between mTORC1 and mTORC2. In our study, compared with rapamycin alone, p-mTOR protein level increased obviously in the co-treatment of rhein and rapamycin in NRK-52E cells exposed to D-gal. Thus, mTORC1, sensitive to rapamycin, may be involved in renal protection. The activation of mTORC2 is intricately linked to PI3K. Several external stimuli, such as immune signals and growth factors, activate PI3K, which catalyzes the transversion of PIP2 to PIP3, and the latter can activate mTORC2 (Liu et al., 2015). Previous studies demonstrated that rhein could protect against oxidative and inflammatory damage through modulating PI3K/Akt signaling (Zhuang et al., 2019; Dong et al., 2022). Therefore, it can be inferred that rhein may influence mTORC2 through regulating PI3K. The regulatory-associated proteins of mTOR (RAPTOR) and the 40-kDa pro-rich Akt-substrate (PRAS40) are the kernels of mTORC1; while the rapamycin-insensitive companion of mTOR (RICTOR), mammalian stressed-activated map-kinase interacting protein1 (mSIN1), and protein observed with RICTOR (PROTOR) are exclusive to mTORC2 (Hung et al., 2012). Further research measuring the levels of these proteins will help clarify the respective contributions of mTORC1 or mTORC2.

Necroptosis depends on the RIPK1-RIPK3-MLKL axis, which is activated by TNF-α, leading to the rupture of the cell membrane. To gain insights into the mechanism by which rhein influences renal aging, we conducted an analysis to find potential targets that are associated with both “rhein” and “aging.” This analysis led to the identification of 23 potential targets that may be implicated in this process. Among the significant pathways in the “rhein–target–pathway” network is the TNF-α signaling pathway. Molecular docking is a bioinformatic modelling technique that simulates molecular recognition computationally (Ferreira et al., 2015). In the present study, the molecular docking patterns of rhein interacting with TNF-α were visualized, with a calculated interaction energy of −8.4 kcal/mol. This suggests that rhein may potentially interact with TNF-α, which could be significant for understanding its role in renal aging. Moreover, untargeted serum metabolomics revealed that rhein exerts a significant impact on the metabolite profiles, and subsequent KEGG pathway enrichment analysis demonstrated prominent enrichment of the TNF-α signaling pathway and its key regulators, strongly implicating TNF modulation as the primary mechanism underlying rhein’s renal protective effects. Xu et al. determined that the TNF-α/TNFR1 signaling cascade plays an essential role in inducing neuronal necroptosis, as evidenced in both cellular cultures and murine models of Alzheimer’s disease (Xu et al., 2021). Additionally, p62 accumulation in response to TNF-α stimulation impairs autophagy flux, activates the RIPK1/RIPK3/MLKL cascade, and results in necroptosis (Xu et al., 2021). In our study, we found that rhein and VE could decrease TNF-α and TNFR1 protein levels in D-gal-stimulated NRK-52E cells and D-gal-induced model rats. Moreover, etanercept as the TNF antagonist, similar to rhein and VE, could downregulate TNF-α, TNFR1, RIPK1, RIPK3, and p-MLKL protein levels, and upregulate caspase-8 and the critical molecules’ protein levels in the mTOR-autophagy signaling pathway in NRK-52E cells stimulated by D-gal. These results strongly suggest that targeting TNF-α is a crucial mechanism by which rhein treats renal injury.

This study provides comprehensive evidence for the renoprotective effects of rhein; however, some limitations should be acknowledged. Firstly, the combined unilateral nephrectomy and D-gal model, while effective, precludes dissection of their individual pathophysiological contributions. Secondly, the central role of TNF-α was primarily validated pharmacologically; genetic approaches, such as TNF-α-specific siRNA or CRISPR-Cas9 knockout, would provide more definitive causal evidence. Additionally, the precise interrelationships among TNF-α inhibition, mTOR activation, and necroptosis suppression warrant future quantitative modeling such as mediation analysis. We also cannot rule out that rhein’s modulation of p-mTOR is partly secondary to its potent ROS-scavenging effect, a possibility that should be disentangled in future studies using combined antioxidant and TNF-α inhibition strategies. Finally, the distinct roles of mTORC1 versus mTORC2 in this context remain to be elucidated. Despite these limitations, our multi-faceted approach converges on a coherent mechanism of action for rhein.

## Conclusion

Our results demonstrated that the renoprotective effect of rhein in oxidative stress-induced renal aging and fibrotic injury was mediated by inhibition of TNF-α-mediated autophagy and necroptosis crosstalk. We found that rhein, similar to VE, was sufficient to attenuate the indicators of renal aging and fibrotic injury by mTOR-mediated autophagy and RIPK1/RIPK3/MLKL-dependent necroptosis. Moreover, rhein might regulate TNF-α-mediated autophagy and necroptosis. Therefore, rhein may serve as a promising therapeutic agent for treating patients with renal aging and fibrotic injury by multiple targets.

## Data Availability

The original contributions presented in the study are included in the article/Supplementary Material, further inquiries can be directed to the corresponding authors.

## Glossary

8-OHdG: 8-hydroxydeoxyguanosine
ALP: alkaline phosphatase
ALT: alanine transaminase
AMPK: AMP-activated protein kinase
AST: aspartate transaminase
BUN: blood urea nitrogen
D-gal: d-galactose
FBS: fetal bovine serum
HBSS: hank’s balanced salt solution
KEGG: kyoto encyclopedia of genes and genomes
LC3: light chain 3
LPS: lipopolysaccharide
MAPKs: mitogen-activated protein kinases
MLKL: mixed lineage kinase-like
mSIN1: mammalian stressed-activated map-kinase interacting protein1
mTOR: mammalian target of rapamycin
PCA: principal component analysis
PCD: programmed cell death
PLS-DA: partial least squares-discriminant analysis
PROTOR: protein observed with RICTOR
PRAS40: 40-kDa pro-rich Akt-substrate
RAPTOR: regulatory-associated proteins of mTOR
RICTOR: rapamycin-insensitive companion of mTOR
RIPK: receptor-interacting protein kinase
ROS: reactive oxygen species
SA-β-gal: senescence-associated-β-galactosidase
Scr: serum creatinine
SD: Sprague–Dawley
TLR4: toll-like receptor 4
TNF-α: tumor necrosis factor-α
TNFR1: TNF-α receptor 1
VE: vitamin E
WB: Western blot
