# Supplementary figures and images for: Rhein protects against renal aging and fibrotic injury by multiple targets through inhibition of TNF-α-mediated autophagy and necroptosis crosstalk
Source: Front Pharmacol. 2026 Jan 26;17:1693000. doi: 10.3389/fphar.2026.1693000 (PMC12883364; doi:10.3389/fphar.2026.1693000)

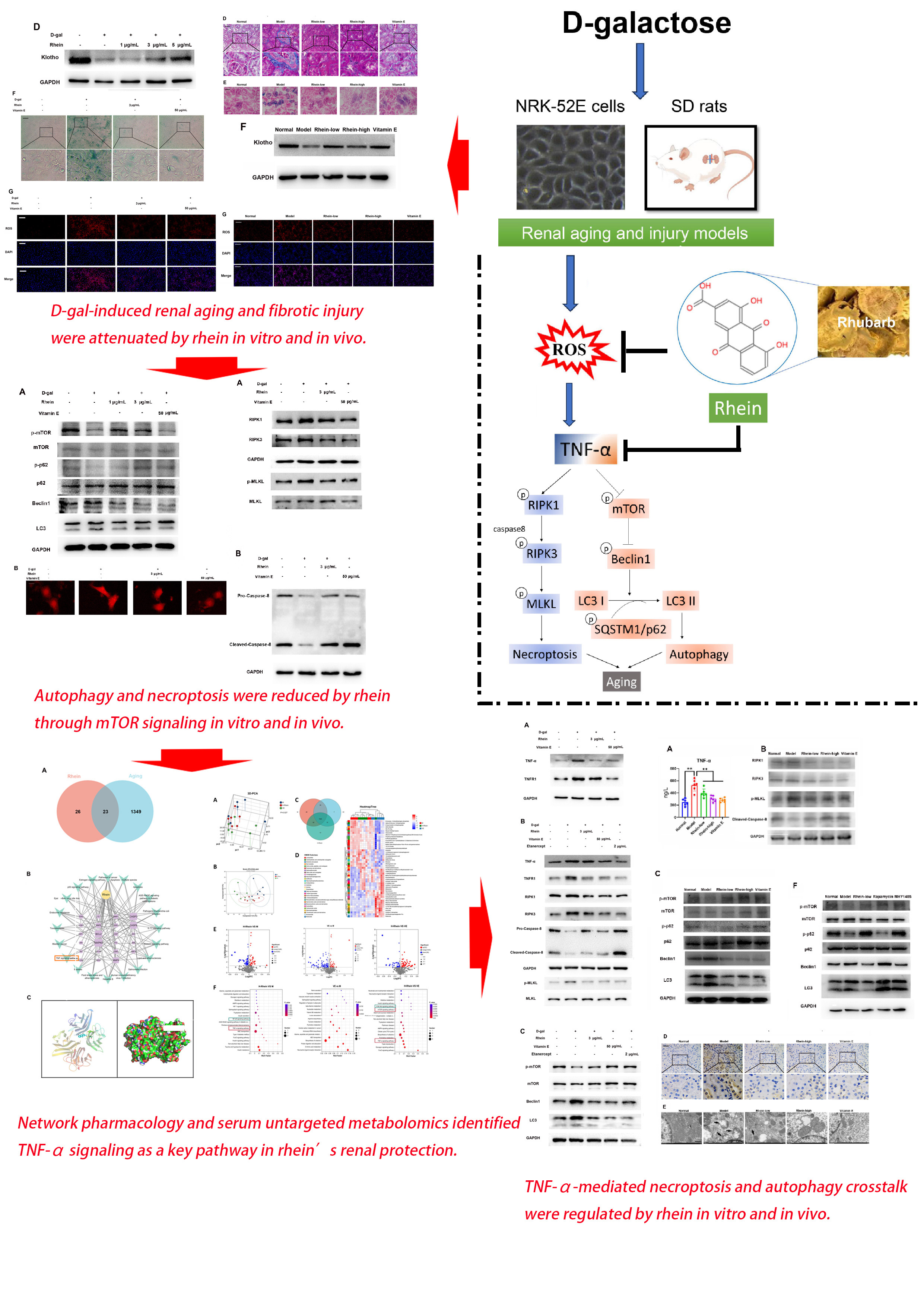

Supplement: Supplementary file 1 [file Image1.jpeg]
