# Supplementary material for: Rhein protects against renal aging and fibrotic injury by multiple targets through inhibition of TNF-α-mediated autophagy and necroptosis crosstalk
Source: Front Pharmacol. 2026 Jan 26;17:1693000. doi: 10.3389/fphar.2026.1693000 (PMC12883364; doi:10.3389/fphar.2026.1693000)
Supplement: Supplementary file 2 [file DataSheet1.pdf]

Supplementary Material

1 Methods

Molecular docking verification

Molecular docking was performed to elucidate the potential interactions between rhein and aging. The 3D structure of target proteins was obtained from the RCSB database (https://www.rcsb.org/), and Open Babel software was used to convert it to the mol2 format. After importing rhein and corresponding potential therapeutic targets into Autodock Tools 1.5.7 for pretreatment and energy optimization, Autodock Vina 1.2.0 was used for molecular docking. The docking score of TNF-α targets and rhein in interaction energy was recorded.

Serum Untargeted Metabolomics

Serum metabolites from Model (M), Rhein-high (H-Rhein) and Vitamin E (VE) groups were extracted with cold acetonitrile:methanol (1:4, v/v) and analyzed by UPLC-MS/MS. Chromatography utilized a Waters ACQUITY UPLC BEH C18 column with a water/acetonitrile gradient. Raw data were processed to identify differential metabolites using criteria of VIP >1 (from PLS-DA model), |Log2FC| ≥1, and P-value <0.05 (Student's t-test). Metabolites were annotated against the KEGG database, and pathway enrichment analysis was performed using a hypergeometric test. Unsupervised PCA and cluster heatmaps were generated for data visualization.

2 **Supplementary Table 1.** Top ten putative protein targets of rhein related to aging

Targets	PDB IDs	Binding energies (kcal/mol)
TNF-α	2AZ5	-8.4
CASP3	3DEH	-8.1
MMP9	5TH6	-8.5
ESR1	3OS8	-8.8
MAPK8	4QTD	-9.4
MMP2	1QIB	-8.4
CASP8	6PX9	-7
MMP1	3SHI	-7.8
ESR2	2Z4B	-9.1
CDK2	2C5Y	-10.7
CASP1	2FQQ	-6.4
ELANE	6E69	-7.2
BCL2	6O0K	-7.6
HNF4A	3FS1	-7.2
MMP13	2OW9	-7.9
CYP19A1	3EQM	-8.5
MMP14	3C7X	-7.3
F2	4HZH	-8.2
INSR	5E1S	-8.8
MME	6SUK	-9.2
LIMK1	5HVJ	-7.8

Supplementary Table 2. Top 20 up- and down-regulated metabolites in different comparisons.

Comparison	Metabolite	VIP	P_value	FC	Regulation
H-Rhein vs. M	Cytidine	2.99	0.0104	2.29	up
	Leu-Ala	3.20	0.0002	2.27	up
	1,1'-(2,6-dimethyl-4-(3-nitrophenyl)-1,4-dihydropyridine-3,5-diyl)diethanone	2.32	0.0103	2.18	up
	1-methylhistamine	2.17	0.0424	2.17	up
	2-acetamido-5-oxo-5-(2-phenylethylamino)pentanoic acid	2.09	0.0159	2.17	up
	Imidazoleacetic acid riboside	2.21	0.0210	2.15	up
	D-Glucaro-1,4-lactone	2.04	0.0088	2.14	up
	N6,N6,N6-Trimethyl-L-lysine	2.07	0.0077	2.13	up
	Cyclo-prolylglycine	1.91	0.0077	2.12	up
	3-Succinoylpyridine	2.00	0.0125	2.11	up
	Leu-Pro	3.87	0.0402	0.31	down
	Orotic acid	4.31	0.0002	0.44	down
	Clethodim	3.80	0.0003	0.45	down
	Phenyllactic acid	2.96	0.0108	0.50	down
	11-Meo-fes	3.10	0.0081	0.46	down
	Tyr-Pro-Phe-Pro-Gly-Pro-Ile	3.15	0.0320	0.46	down
	Citraconic acid	2.76	0.0323	0.46	down
	5-Acetamido-4-oxohexanoic acid	2.79	0.0429	0.46	down
	Hippuric acid	2.93	0.0176	0.47	down
	4,5-Dihydroxy-4-(2-hydroxyethyl)cyclohex-2-en-1-one	2.70	0.0173	0.48	down
VE vs. M	Afalanine	2.71	0.0045	2.38	up
	N-(2-formyl-3-chlorophenyl)anthranilic acid	2.23	0.0336	2.29	up

	N-Acetyl-D-phenylalanine	2.17	0.0313	2.26	up
	P-Coumaraldehyde	2.01	0.0340	2.26	up
	6-Hydroxyhexanoic acid	2.22	0.0296	2.24	up
	N-Lactoylleucine	2.43	0.0199	2.22	up
	4'-Nitrophenyl-2-acetamido-2-deoxy-beta-glucopyranoside	2.03	0.0237	2.21	up
	Uridine diphosphate-N-acetylglucosamine	2.11	0.0037	2.21	up
	Pcera-1	2.39	0.0124	2.20	up
	3-hydroxycinnamic acid	2.22	0.0305	2.19	up
	Hypoxanthine	3.90	0.0005	0.37	down
	Isopimpinellin	3.61	0.0005	0.40	down
	KAPA	3.23	0.0146	0.42	down
	Sesamose	2.99	0.0335	0.42	down
	Leu-Val	3.02	0.0094	0.45	down
	Leukotriene D4	3.05	0.0063	0.46	down
	1-Hydroxypyrene	2.61	0.0428	0.47	down
	Trp-Asp	2.79	0.0158	0.45	down
	Dl-4-hydroxyphenyllactic acid	2.80	0.0428	0.45	down
	2-[2-(2-pyridyloxy)ethoxy]pyridine	2.80	0.0152	0.45	down
H-Rhein vs. VE	2-Hydroxybutyric acid	1.20	0.0092	3.06	up
	4-(2-Aminophenyl)-2,4-dioxobutanoic acid	1.34	0.0024	3.05	up
	3-Coumaric acid	4.21	0.0036	3.00	up
	Linoelaidic acid	1.01	0.0384	2.98	up
	Daphniphylline	1.02	0.0159	2.98	up
	Pc(22:6/0:0)	1.04	0.0362	2.98	up
	1-(6-((3-Methoxyestra-1,3,5(10)-trien-17-yl)amino)hexyl)-1H-pyrrole-2,5-dione	1.12	0.0432	2.98	up
	4-O-alpha-D-	1.01	0.0417	2.98	up

	Galactopyranuronosyl-D-galacturonic acid				
	GPCho(18:1/18:1)	1.12	0.0399	2.98	up
	5-hete lactone	1.16	0.0435	2.98	up
	AB-Pinaca	2.80	0.0253	0.36	down
	Diketogulonic acid	2.67	0.0082	0.39	down
	4-ethoxy-6,7-dimethoxyquinazoline	2.48	0.0405	0.41	down
	Thr-Ile	2.50	0.0217	0.41	down
	Cyclo(Pro-Val)	2.52	0.0185	0.41	down
	N-acetyl-l-tyrosine	2.33	0.0262	0.41	down
	Melatonin	2.25	0.0443	0.42	down
	15-Demethyl plumieride	2.22	0.0455	0.42	down
	Riboflavin	2.65	0.0074	0.42	down
	4-hydroxy Nonenal Glutathione	2.06	0.0403	0.43	down

Abbreviations: VIP: Variable Importance in the Projection; FC: Fold Change.
